# Initial experience with hysteroscopic tubal occlusion (Essure^®^)

**DOI:** 10.1590/S1679-45082016AO3717

**Published:** 2016

**Authors:** Daniella De Batista Depes, Ana Maria Gomes Pereira, Umberto Gazi Lippi, João Alfredo Martins, Reginaldo Guedes Coelho Lopes

**Affiliations:** 1 Hospital do Servidor Público Estadual “Francisco Morato de Oliveira”, São Paulo, SP, Brazil.

**Keywords:** Sterilization, tubal/methods, Hysteroscopy/methods, Contraception, Ambulatory surgical procedures

## Abstract

**Objective:**

To evaluate results of early tubal occlusions performed by hysteroscopy (Essure^®^)_._

**Methods:**

This prospective study included 38 patients, 73.7% of them were white, mean age 34.5 years, they have had on average 3 pregnancies and 2.7 of deliveries. A total of 86.8% of patients previously prepared the endometrium. All procedures were carried out at outpatient unit without anesthesia**.**

**Results:**

Insertion rate of the device was 100% at a mean time of 4 minutes and 50 seconds. Based on the analogical visual scale, average pain reported was three, and 55.3% of women did not report pain after the procedure. After 3 months, 89.5% of patients were very satisfied with the method. Simple radiographs of the pelvis showed 92.1% of topical devices, and one case of unilateral expulsion had occurred. A four years follow-up did not show failure in the method.

**Conclusions:**

Tubal occlusion through hysteroscopy at outpatient unit and without anesthesia was a quickly and well-tolerated procedure. No serious complications were seen, the success rate was high, and patients were satisfied.

## RESUMO

**Objetivo:**

Avaliar os resultados das primeiras oclusões tubárias realizadas pela via histeroscópica (Essure^®^).

**Métodos:**

Estudo prospectivo com 38 pacientes, sendo 73,7% caucasianas, com média de idade de 34,5 anos, e com 3 gestações e 2,7 partos em média. Do total, 86,8% das pacientes fizeram preparo prévio do endométrio. Todos os procedimentos foram ambulatoriais e sem anestesia.

**Resultados:**

A taxa de inserção do dispositivo foi de 100%, com tempo médio de 4 minutos e 50 segundos. Segundo a Escala Visual Analógica, a dor média obtida foi de três, e 55,3% das mulheres não referiram qualquer dor após o método. Ocorreu um caso de reflexo vagal e 89,5% das pacientes retornaram às atividades normais no mesmo dia. Após 3 meses, 89,5% das pacientes encontravam-se muito satisfeitas com o método. A radiografia simples da pelve evidenciou 92,1% de dispositivos tópicos, ocorrendo um caso de expulsão unilateral. Após 4 anos de seguimento, não houve falha do método.

**Conclusão:**

A oclusão tubária por via histeroscópica em regime ambulatorial e sem anestesia foi um procedimento rápido, bem tolerado, isento de complicações graves e com alta taxa de sucesso e satisfação das pacientes.

## INTRODUCTION

Female sterilization by tubal ligation or occlusion is the most effective and used method for family planning worldwide.^(1)^ This technique have been changed progressively by the introduction of minimally invasive surgery. In developing countries the minilaparotomy remains the most common procedure, but in developed countries interval laparoscopic sterilization is most the common technique.^(2)^

Laparoscopic ligature is efficient, but invasive and with possible anesthesia and surgical risks, such as vascular and bowel injury.^(3)^Definitive and ideal contraceptive method would be highly efficient and cause minimal complications. Transcervical access constitutes an appealing alternative for transabdominal access, and it does not require incisions, general anesthesia, and avoids the risk of accessing the abdominal cavity. However, development of safety and effective transcervical methods seems to be difficult.^(4)^

Essure^®^, Bayer AG (Leverkusen, Germany) was the first mechanical device approved in 2002 by Food and Drug Administration (FDA) for transcervical sterilization. This device was approved in 2009 by the Brazilian Health Surveillance Agency (ANVISA).^(5)^

The system includes an intratubal device, a release system and one catheter to access each tube by transcervical route. The microdevice is a dynamically expanding coil made up of internal ring of stainless steel involved by external ring made of nickel titanium, which expansiveness maintains the device into the uterine-tubal junction during the period required to occur the fibrosis. Polyester fibers are around the central structure and may cause reaction to surrounding tissue, followed by fibrosis, therefore, promoting an irreversible fallopian tube occlusion. This process occurs in approximately 3 months and, during this period, the woman must maintain the use of oral contraception.^(4)^

The device has 26 spirals with 4cm length and 0.8mm diameter, and it expands to 1 to 2mm in diameter. The insertion consists of catheter passing through the tubes with the Essure^®^ system inside it during the hysteroscopy. Placement is considered successful when three to eight spirals remains visible in the uterine cavity.^(6)^

Review after implantation of the device is considered the final part of the procedure and three months are required before check if the implant is on the pelvis and at adequate position. In the United States, a hysterosalpingography (HSG) is requested, but in other countries a simple radiography of pelvis or ultrasonography is needed. If the device is satisfactory placed in uterine-tubal junction, the patient can stop the other contraception methods. If position is unsatisfactory, HSG is needed.^(2)^

After 5 years, Essure^®^ system showed efficacy of 99.74%_._^(5)^Most of gestation occurred because the patient did not follow medical guidance of use other contraceptive method for 3 months after placement or did not return to confirm the occlusion. There were also some mistakes in tests interpretation, and some patients were already pregnant at the time of placement.^(5)^Veersema reports success in placement of 95% to 99% of cases at outpatient unit.^(2)^Complications associated with the method are quite rare and include inadequate placement (poor placement), device migration, unintended pregnancies, pain, infection and nickel allergy. Uterine or tubal perforation can result in pregnancy or chronic pelvic pain.^(7)^Because this is an irreversible method, the main contraindication is for patient who is unsure of definitive contraception. Other active or recent gynecologic infection, the malignant gynecologic tumor and current use of corticosteroids, if the medication cannot be stopped for 3 months. After delivery or pregnancy interruption a period of 6 weeks should be wait for insertion of the device.^(6)^

Essure^®^ has the following advantages compared with laparoscopic tubal ligation: it does not require any incision, the insertion is done on an outpatient basis, it does not require anesthesia, time waste in the procedure is minimal, and patients are discharged immediately after the procedure and can return to daily activities on the same day after the placement.^(6)^

## OBJECTIVE

To evaluate acceptance of tubal occlusion method by hysteroscopy done on an outpatient basis without anesthesia, and also assess device insertion rate, time spend in the procedure, complications, pain after placement, time needed to return for normal activities, tubal occlusion rate, patient’s degree of satisfaction, pregnancy rate after 4 years of follow-up.

## METHODS

This prospective study was carried out from February 2009 to July 2010 at the hysteroscopy unit of the *Hospital do Servidor Público “Francisco Morato de Oliveira”*. We included 39 patients. Of them, 73.7% were white with mean age 34.5 years, and have had on average 3 pregnancies and 2.7 deliveries. A total of 86.8% of patients had previously prepared the endometrium. Patients included chosen definitive contraceptive method after receive information about other options/methods at Family Planning Sector. All patients were informed about available methods of tubal occlusion (laparatomic, laparoscopic and hysteroscopic). Those who decided to do the latter method signed the consent form. This study was approved by Institutional Ethics and Research Committee, number CAAE: 0095.1.338.000-10.

All patients received anti-inflammatory drugs (Ibuprofen 600mg) and benzodiazepines drugs (diazepam 10mg) one hour before the procedure to avoid spasm of uterine tubes.

Device insertion occurred during ambulatory hysteroscopy beginning with vaginoscopy, without the use of speculum or Pozzi forceps to traction the cervix. The procedures entails catheter passing through the tubes with the Essure^®^ system inside it. This catheter is introduced through (1.7mm) operative channel 5F of Bettocchi^®^ hysteroscopy (Karl Storz, Germany). We used saline solution for uterine distension, and no anesthesia during the procedure. The procedure was carried out in first phase of the cycle or during the use of contraceptive drugs, for good visibility of uterine cavity and tubal ostiums. Those procedures performed during the use of levonorgestrel intrauterine system did not show any additional difficult because this device does not prevent tubal catheterization.

Pain was evaluated by patients immediately after the device placement based on Visual Analogue Scale (VAS). This measure comprised of line of 10cm with anchors in both extremities. One extremity is identified as “no pain”, and the other “pain as bad as it possibly be”. This instrument has been considered sensible, simple, reproducible and universal.^(8)^ Because this procedure recovery is immediately, no resting was need after the procedure at the outpatient unit.

After 30 days, in the first return consultation, patients reported the presence or absence of pain just after the hysteroscopy and in the subsequent days, in addition, they also reported time needed to return to daily life activities.

After 3 months of Essure^®^ insertion, a simple radiographic of pelvic region was done. If the device was satisfactory placed in the uterus – tubal junction, the occlusion was considered definitive. If the position was unsatisfactory, an HSG was requested. At this time, patients’ satisfaction with the method was assessed. Four years after insertion, the investigator called patients to collect information about possible failures of the method. Variables included were age, number of pregnancies, parity, type of delivery, formal education, endometrial preparation, pain during and after the procedure, duration of the procedure, time needed to return to daily activities, satisfaction with the method, position of the device on simple radiographic of the pelvis and presence of tubal occlusion in the HSG.

Pain during the procedure was stratified into three categories: 0 to 4 (no pain or minimal pain), 5 to 7 (moderate pain) and 8 to 10 (intense pain). All information and results were analyzed using the statistical software Epi Info™ 3.5.2 for Windows, with descriptive calculation of mean and standard deviation for quantitative variables and frequency of qualitative variables. Univariate analysis was done using the Pearson’s correlation test. The p<0.05 was considered statistical significant in a confidence interval of 95% (95%CI).

## RESULTS

We included 38 patients with mean age 34.5 years, and 73.7% of participants were white. Of them, 28.9% had finished elementary school, 39.5% had attended high school, and 31.6% graduated from college. Number of pregnancies ranged from one to nine, mean of three gestations, and mean of 2.7% deliveries.

A total of 86.8% of patients had previous endometrial preparation. Of them, 78.8% used oral combined contraceptives, 15.2% used oral progestogen, and 3.0% used combined injectable contraceptives. Only one patient used the levonorgestrel intrauterine system. Endometrial preparation did not change the time required for the device placement.

All devices were successful placed bilaterally, procedural time was on average 4.5 minutes. Pain scale ranged from zero to 10, mean of three. Patients reporting no pain or mild pain were 68.4%, 23.7% reported moderate pain, and 7.9% reported intense pain. There was no association between pain scale and formal education, type of delivery, time required for the device placement, however, a correlation was seen related to the number of pregnancies and deliveries (Table 1).

**Table 1:** Correlation between clinical data and pain intensity during the procedure

Clinical data	95%CI	p value*
Pregnancies	-1,155- -0,139	0,014
Parity	-1,209- -0,015	0,045
No endometrial preparation	-5,304- 0,055	0,055
Cesarean section	-0,258-3,681	0,086
Age	-0,052-0,280	0,173
Formal education	-1,173-2,891	0,397
BMI	-0,129-0,268	0,483
Duration of the procedure	-0,437-0,655	0,688

*p value: Pearson’s statistical significance.

95%CI: 95% confidence interval; BMI: body mass index.

One patient had vagal reflex that stopped rapidly after the use of sublingual atropine. No pain after the procedure was reported by 55.3% of patients. Among those who remained in pain, 39.5% reported pain lasting for 2 days and, in 5.3% the pain lasted for more than 2 days.

Time to return for daily activities ranged from zero to 5 days, and 89.5% of patients returned to daily routine on the same day. At return consultation, after 3 months, 89.5% of patients were very satisfied, 10.5% were satisfied and no patient was disappointed with the method.

Radiographies showed that 92.1% of devices were topic and tubes were considered obstructed. In 7.9% of cases there were uncertainty about Essure^®^ localization, and the HSG showed bilateral tubal obstruction in 75% of patients. In one case, we observed unilaterally patent tube because of the device migration to the pelvis. After 4 years of sterilization, no failure to prevent pregnancy was reported.

## DISCUSSION

Although Essure^®^ is a new method in Brazil, more than 750,000 woman worldwide are using this method, which cause a conceptual revolution in permanent birth control procedure.

Success rate in first attempt of insertion reported by many authors ranges from 81 to 98%.^(4)^ In our study success rate was 100%, probably because of our small sample.

The main reasons of non-insertion of a device are poor visualization of ostiums, and when the procedure is done in first phase of menstrual cycle or previous endometrial preparation with hormones. We attempted to prevent tubal spasm before the procedure based on report of a number of authors by using anti-inflammatory drugs and benzodiazepines.^(4,6)^

All procedures were carried out at the outpatient units. Evidences show that efficacy is the same when compared to procedures performed at surgical center_._^(5)^ Pain is the main concern of patients, we used the same approach described by Veersema, which is previously inform that discomfort would be similar to menstrual cramps, this information helped patients to become less concerned.^(2)^

Vaginoscopy technique used in all cases along with low diameter of optics have caused lower discomfort for patients, not requiring the use of anesthesia. The vaginoscopy was not used, and mean pain reported was 3.0, based on a visual analogical scale from zero to 10cm. Previous studies in hysteroscopy at outpatient unit showed pain ranging from 3.2 to 4.7, using the same pain scale.^(3)^According to Veersema, pain is related to duration of the procedure.^(2)^ The procedures last, on average, for 4 minutes and 50 seconds, which probably cause high tolerability and patients’ satisfaction.

The complication of the procedure was vagal reflex (2.6%). Other studies reported 3.7% of same reaction, but recovery was immediate after use of atropine.^(6)^

Arjona et al. carried out 1,630 procedures at outpatient unit without anesthesia, and 80% of women returned to daily activities on the same day. In our study 89.5% of participants returned to daily activities on the same day.^(9)^

Simple pelvic radiography was used as the control test 3 months after the procedure. In three cases, because of uncertainty of correct positioning, the HSG was done. In one case, there was migration unilaterally of the device to pelvis, being the device removed through laparoscopy. Aparicio-Rodríguez-Miñon et al. studied 517 patients and found two expulsion of Essure^®^ from uterine cavity and one bilateral migration of the device to pelvis, which was identified and removed during laparoscopic tubal ligation.^(6)^ Migration of the device for pelvic cavity is rare, its incidence reported is 0.1%.^(7,10)^

All patients completed the 3 months follow-up. When patients was inquired about satisfaction with the method, 89.5% were very satisfied, 10.5% satisfied, and no patient was disappointed with the method. Another study evaluated satisfaction rate during return consultation after 3 months and described that 94% of patients were very satisfied with the procedure, 6% had some type of satisfaction and no patients were dissatisfied.^(9,10)^

Recent literature reviews show unintended pregnancy rate after Essure^®^ placement as almost inexistent.^(6)^ No patients in our study get pregnant after a 4 year follow-up.

## CONCLUSION

Tubal occlusion by hysteroscopy at outpatient unit and without anesthesia was a quick and well-tolerated procedure. We did not observe severe complications. Success rate and patients’ satisfaction were high.
